# Identifying Pathogenicity Islands in Bacterial Pathogenomics Using Computational Approaches

**DOI:** 10.3390/pathogens3010036

**Published:** 2014-01-13

**Authors:** Dongsheng Che, Mohammad Shabbir Hasan, Bernard Chen

**Affiliations:** 1 Department of Computer Science, East Stroudsburg University of Pennsylvania, East Stroudsburg, PA 18301, USA; 2 Department of Computer Science, Virginia Polytechnic Institute and State University, Blacksburg, VA 24061, USA; E-Mail: shabbir5@vt.edu; 3 Department of Computer Science, University of Central Arkansas, Conway, AR 72035, USA; E-Mail: bchen@uca.edu

**Keywords:** genomic islands, pathogenicity islands, computational methods, genomic signature, mobility gene, virulence factors, pathogenicity island database

## Abstract

High-throughput sequencing technologies have made it possible to study bacteria through analyzing their genome sequences. For instance, comparative genome sequence analyses can reveal the phenomenon such as gene loss, gene gain, or gene exchange in a genome. By analyzing pathogenic bacterial genomes, we can discover that pathogenic genomic regions in many pathogenic bacteria are horizontally transferred from other bacteria, and these regions are also known as pathogenicity islands (PAIs). PAIs have some detectable properties, such as having different genomic signatures than the rest of the host genomes, and containing mobility genes so that they can be integrated into the host genome. In this review, we will discuss various pathogenicity island-associated features and current computational approaches for the identification of PAIs. Existing pathogenicity island databases and related computational resources will also be discussed, so that researchers may find it to be useful for the studies of bacterial evolution and pathogenicity mechanisms.

## 1. Introduction

In past decades, researchers have used comparative genome analyses to study bacterial evolution. When studying genome sequences, researchers discovered that some genes were missing in these genomes, but were present in their phylogenetically closely-related genomes. On the other hand, some genes were present in some genomes but were missing in the genomes of the same species. It has been now generally understood that gene loss and gene gain in the genomes are the driving forces for genome evolution [1].

The cause of gene loss might be related to the change of living niches, where the existence of such genes in the host genome may confer the fitness cost [2]. Gene loss could also be a positive selection. For instance, the loss of gene *cadA* in Shigellae results in the loss of its product [3]. The absence of this product can trigger the production of plamid-encoded virulence factors, and, thus, making Shigellae more pathogenic.

Gene gain in bacterial genomes might also be selected by the change of their living environment, and acquiring genes make them more selective. For instance, some marine Actinobacteria *Salinispora tropica* and *Salinispora arenicola* were discovered to harbor genes associated with secondary metabolite biosynthesis to adapt their living niches [4]. The process of transferring the genes from alien genomes into the host genomes is known as *horizontal gene transfer*, which is in contrast to *lateral gene transfer*, where gene transfers occur within the host genomes. Due to the fact that horizontally transferred genes have their alien origin, such regions are known as *genomic islands* (GIs).

The concept of GIs was from Pathogenicity Islands (PAIs), which was first created by Hacker and his colleagues [5]. They used it to describe a functionality of a genomic region of *Escherichia coli* that harbors clusters of virulence factors that can be simultaneously deleted. Later on, researchers found more clusters of genes with different functionalities, including groups of genes which encode antibiotic resistance, also known as *antibiotic resistance islands*, or some other gene group that encode adaptive metabolic properties such as phenolic compound degradation, also known as *metabolic islands*.

The studies of GIs are very important to biomedical and bioinformatics research. This is because we can use GIs to explain why some strains of bacteria within the same species are pathogenic while others are not, why some specific species could survive in extremely critical living environment while others do not; we can also use GIs to understand the functionalities of a bacteria and genome evolution. Therefore, the identification of GIs represents one of crucial tasks for genome evolution and gene transfer mechanism studies.

In this review, we will focus on the identification of PAIs, one of the most important GI groups. We will look into PAI-related features, and then review current available computational approaches for PAI identification. We will highlight some of important PAI databases and related resources for community access. It should be noted that most of computational tools and database available are for genomic islands in general, meaning that they provide not only predicted PAIs, but also other groups of genomic islands, such as resistance islands or metabolic islands.

## 2. PAIs-Related Features

**Figure 1:**
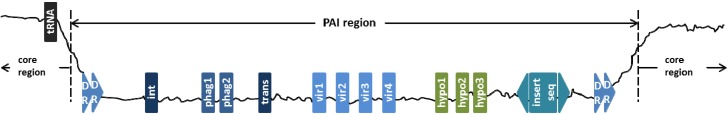
A schematic view of a pathogenicity island with associated features. The PAI region has biased sequence composition. The PAI regions are associated with virulence genes (vir1, vir2, vir3, and vir4), phage-related genes (phag1 and phag2), mobile genes (int and trans), hypothetic protein genes (hypo1, hypo2, and hypo3), insertion sequence elements, direct repeats, and tRNA gene.

When comparing the genomic region of PAIs and the remaining parts of the host genome, we can usually find that PAIs have their own genomic characteristics such as containing mobility genes, containing virulence genes, and having their own sequence signature. Figure 1 illustrates a schematic view of a PAI. The PAI associated features and corresponding measurement methods summarized in Table 1.

**Table 1:** A list of Pathogenicity Islands (PAI)-associated features and measurement methods.

PAI-Associated Features	Feature Measurement Methods
Different genomic sequence signature	Compute G+C content, GC-skew, codon usage, or other sequence signature tools
Presence of virulence factors	Search through virulence factor database such as VFDB
Presence of mobility genes (integrases, transposes)	Search through NCBI-nr/nt, UniprotKB, Pfam or COG database
High percentage of phage-related genes	Search through NCBI-nr/nt, UniprotKB, Pfam or COG database
Presence of tRNA genes	Use tRNA gene search tool of tRNAscan-SE
High percentage of hypothetic protein genes	Search through NCBI-nr/nt, UniprotKB, Pfam or COG database
Presence of direct repeats	Use repeat finder software REPuter
Presence of insertion sequences	Search through ISfinder database

### 2.1. Genomic Sequence Signature

In general, each genome has its unique genome signature, which can be measured by G+C content, dinucleotide frequencies (or other *k*-mer frequencies), and codon usage. This is because PAIs were originally transferred from other pathogenic bacteria, plasmids, or phages, and, thus, the genomic sequence structure of PAIs is different from that of the rest of host genome.

#### 2.1.1. G+C Content and GC-Skew

The G+C (%) contents (*i.e.*, the percentage of guanine and cytosine bases) in PAIs are often different from that of the host organisms. For instance, the G+C content of the Uropathogenic *E. coli* core genome was 51%, while the G+C content was 41% in PAI I, II, IV, and V [6,7,8]. In the genome of Enteropathogenic *E. coli*, the G+C content of PAI of LEE was only 39% [9]. G+C content differences between PAIs and the core genomes have also been found in other genomes, such as *D. noddosus* [10], *H. pylori* [11], and *Y. pestis* [12].

A slightly different measure can also be used for measuring the difference between PAI and core region. For instance, a large scale comparative genomic analyses of 1,088 bacterial and Archaeal genomes showed that PAIs were anchored around switch sites of GC-skew (sGCS), which was measured by [G−C]/[G+C] [13].

#### 2.1.2. *k*-Mer Frequency

The measurements of dinucleotides or high-order oligonucleotide frequencies have been increasingly used [14]. Theoretically, the higher-order measurement used, the more accurate to differentiate two genomes, given the assumption that the genomic region for measurement is long enough to evaluate all combinations of oligonucleotide patterns (or words). For instance, if 6-mer frequency is used, then there will be 4^6^ = 4,096 words, and, thus, a genomic region with at least several *kb* is required if 6-mer frequency is used. We have seen several approaches such as AlienHunter [15] and Centroid [16] that used *k*-mer frequencies to predict island regions.

#### 2.1.3. Codon Usage

Codon usage is another useful feature to tell the differences between two genomes. Generally speaking, each genome has its own preferred codon usage, and thus the codon usage in a genome region will be significantly different than the rest of host genome if this region was transferred from outsider. SIGI-HMM software uses codon usage bias to predict GIs [17].

#### 2.1.4. Caveat

While PAIs have skewed sequence composition, highly expressed genes (HEGs) (including ribosomal related genes, chaperonin genes, transcription and termination factor genes, energy metabolism genes, recombination and repair genes, and electron transport genes) may also have codon usage bias and dinucleotide bias [18]. In this scenario, using sequence composition information only to detect islands in pathogenic bacteria will lead to the problem of *false positives* (*i.e.*, predicted PAIs might actually be HEGs).

On the other hand, it is possible that the donor and recipient organisms have similar sequence composition, thus, making it difficult to dig out those real PAIs sporadically distributed in the core genome. Furthermore, even the donor and recipient organisms have different sequence compositions, it is very likely that the PAI region will be eventually ameliorated, a process that makes the sequence composition (or codon usage) of the alien genomic region (*i.e.*, PAIs) be similar to that of the core genome, so that the integrated region can be adapted to enhance expression [19]. A recent large scale genomic study of 1,088 bacterial and Archaeal genomes has shown the newer acquired PAIs were closer to sGCS than the older ones, implying that the older PAIs are in the process of amelioration [13]. In this scenario, using sequence composition information only will lead to the problem of *false negatives* (*i.e.*, the actual PAIs may not be discovered easily).

### 2.2. Virulence-Associated Genes

Another important property of PAIs is that PAIs contain virulence-associated genes. Depending on the environment that bacteria live in, the proteins encoding virulence-associated genes in PAIs can include the following categories: (a) Adhesins, which are cell-surface components that facilitate bacteria adhere to eukaryotic cells; (b) Type III and IV secretion systems, which have needle-like structures that detect the presence of host organisms, and secrete effector proteins into the host cell; (c) Invasins, which facilitate bacteria to invade eukaryotic epithelial cells; (d) Toxins, which can be exotoxins, proteases, lipases, and enterotoxins; and (e) Iron uptake systems. The details of virulence factors existed in PAIs of pathogenic bacteria can be found in other reviews [20,21,22].

In a recent large-scale analysis study between virulence factors and GIs [23], the researchers found that virulent factors were disproportionately found in GIs. Furthermore, Offensive virulence factors, such as toxin, type III secretion system, and type IV secretion system, were found more in pathogenic genomes than in non-pathogenic genomes. On the other hand, other categories of virulence factors such as motility, antiphagocytosis, and iron uptake were found more in non-pathogenic genomes than in pathogenic genomes. These findings indicate that PAIs contain manly offensive virulence factors rather than other categories. Virulence factors in any genomic sequence can be identified through a BLAST search against virulence factor database [24], or virulent factor prediction tools, such as VirulentPred [25].

### 2.3. Mobility Genes

Two kinds of mobility genes, integrase gene and transposase gene, are often found in PAIs. The integrase gene is involved in the integration, recombination, or excision of mobile elements. Transposase is an enzyme that helps the movement of transposons from one region to another. The mechanisms of how alien genes are transferred, stabilized, or excised from the host genome, or how mobility genes get involved in such processes can be found in other reviews [26]. The identification of the mobility genes can be done through searching Pfam protein database using HMMER3 [27,28].

### 2.4. Phage-Related Genes

High percentage of phage-related genes has been found in PAIs [29,30]. In actuality, phage transduction and prophage integration are the major mechanisms of horizontal gene transfer in prokaryotes [31]. The food pathogen *E. coli* O156:H7 strain Sakai has been discovered to contain around 16% prophage of its own total genome sequence [32]. The identification of the phage-related genes can be done through searching Pfam protein database using HMMER3 [27,28].

### 2.5. Transfer RNA

Many PAIs are flanked by tRNA genes, and other elements, such as integrases and insertion sequence elements. tRNAs may be involved in insertion process at the insertion points when PAIs are inserted into the host genome. It is generally considered that not all tRNAs loci are targeted for insertion sites, but with some bias. For example, in a study of 328 tRNA orthologs from four genomes, researchers found that there were only 18 tRNA loci for insertion sites [33]. Further, tRNA loci bias may be different in different genomes. One study showed that preferred insertion site were tRNA-Arg, tRNA-Leu, tRNA-Thr, and tRNA-Ser, but few at tmRNA sites [34]. In another study of 168 islands, the most frequently targeted tRNA loci were tmRNA and tRNA-Ser [35]. While finding tRNA genes can be achieved using the software tool of tRNAscan-SE [36], it can be difficult to find tRNA loci for insertion sites, given that different genomes have their preferred tRNA loci.

### 2.6. Hypothetical Protein Genes

Compared to the core genome, PAIs have high percentage of hypothetical protein genes (*i.e.*, proteins with unknown function) [37]. This can be explained that the donors (might also include plasmids or pro-phages) might have not been cultured and sequenced yet, and the functions remain to be determined. Hypothetic protein genes can be obtained by finding open reading frames (ORFs) with gene-finding programs, such as GeneMark [38] and Glimmer [39], and then excluding proteins with known functions, which be searched against NCBI-nr/nt [40], UniprotKB [41], Pfam [28], or COG database [42].

When measuring inter-genic distances (*i.e.*, the distances between any two adjacent genes) between the known island regions and the core regions, Wang *et al*. [43] found that, on average, island regions had longer inter-genic distance than those of the core genome. Island regions might bring some alien genomic sequences that have yet to be predicted as ORFs, but involved in some unclear activities such as transposon-related activity.

### 2.7. Direct Repeat

PAIs are usually flanked by direct repeat (DR) sequences, in which each DR is 16–20 bp long with nearly perfect sequence repetition. The DR sequences might be generated when mobile elements were integrated into the host genome, and act as target sequences for the excision of mobile elements from the host genome [44]. DRs can be detected by genomic sequence analysis software tool of REPuter [45].

### 2.8. Insertion Sequence (IS) Elements

PAIs may also contain IS elements, which are usually flanked by inverted repeat sequences. Unlike DR sequences (usually flanked by PAIs), IS elements are very often part of PAI segment. IS elements may medicate DNA rearrangements by transpositional events, and act as the target sequences for the excision of mobile elements in the host genome. IS elements can be identified by searching ISfinder database [46].

## 3. Computational Identification of PAIs

Currently there are a number of island prediction approaches, which are generally based on either (i) comparative genomics to find unique regions which are absent in several related isolates or (ii) sequence features and sequence composition differences. The first category can be considered as comparative genomics based approach, and the second one can be termed as sequence composition based approach.

### 3.1. Comparative Genomics Based Approach

**Figure 2:**
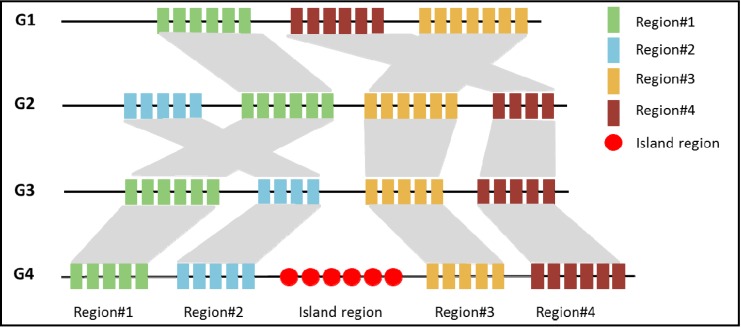
A schematic view of genomic region alignment in the comparative genomic based approach for island prediction. Three phylogenetically closely-related reference genomes (G1, G2, and G3) are shown here for the detection of island region in the query genome (G4).

Comparative genomics-based approach compares the incongruence of the gene tree *versus* its associated species tree [47]. A “gene tree” is the phylogeny of alleles or haplotypes for any specified stretch of DNA [48]. Several kinds of computational tools have been developed based on the rule that states that the genomes of closely related species should be highly assumed to share similar preferences and signatures. Therefore, if a genomic sequence of one species contains some special signatures that other species do not have, it is highly recommended that this genomic sequence has a foreign origin. The comparative genomic-based approach consists of three general steps: (1) Collecting all genome sequences from closely related species for a query genome; (2) Aligning these genome sequences together; and (3) Considering those gene segments present in the query genome but not present in others to be islands. Figure 2 shows a schematic diagram of comparative genomic-based approach for island prediction. Below, we describe three comparative genomics approaches for predicting GIs in general.

IslandPIck [49] is one of the popular comparative genomic-based sequence approaches. This approach starts with using a distance function to measure the phylogeny relatedness of the reference genomes with the query genome using a tool named CVTree [50], and then picks appropriate genomes for genome alignment. After the genome selection step, IslandPick uses the Mauve [51] program to pairwise genome alignments and identify unique regions of the query genomes, which are considered to be GIs. It uses Mauve again to do multiple genome alignments and identifies the genomic regions that are common to all genomes, which are considered as non-GIs.

MobilomeFINDER is another tool that uses comparative genomics-based approach for GI prediction [33]. The basic idea of this approach is quite similar to that of Islandpick, but it includes the information of tRNAs, as tRNA has been found to be involved in GI insertion process. However, the disadvantage of this approach is it may lead to false negative GI prediction. This is because not all GIs contain tRNAs as insertion points, therefore, MobilomeFINDER will miss some of GIs without tRNA present.

MOSAIC [52,53] is another comparative genomic approach, and the authors built an online database that provides the alignment results of bacterial genomes. The genome segmentation process includes four major steps: (1) selecting related genomes using Mummer3 [54] and Multiple Genome Alignment (MGA) [55]; (2) aligning genomes using MGA; (3) generating backbone (*i.e.*, conserved regions) and loops (strain-specific segments); and (4) database integration. This database considers variable regions (*i.e.*, loops) in the alignment results to be GI regions. There is a user-friendly web interface that facilitates the browsing and downloading of these GI regions, which illustrates the important properties of these regions. Such segmentation results along with the visualization of these bacterial genomes are useful to the researchers for functional analysis. 

The advantage of comparative genomic approaches is that it is easy to identify the difference between closely related genome sequences, which are supposed to share same gene contents and signatures. The disadvantage, however, is that we do not have enough close-related genomes available for some query genome, and, thus, this approach cannot be applied to any sequenced genome. Another disadvantage of this approach is, most of the computation tools need manually adjustment and selection, which is hard to perform and control as it may lead to inconsistent selection criteria due to the unfamiliarity of different genome structure [33].

### 3.2. Sequence Composition Based Approach

Theoretically, all genomic regions inside the host genome are supposed to share same genomic signature. If a piece of genomic sequence has been detected with different gene signature or contents, it is highly recommended that this region is horizontally transferred from other sources. Sequence composition approach is based on this concept that enables us to make genomic region comparison within one single genome to identify special signatures. These genomic signatures include G+C content, dinucleotide frequencies, codon usage, mobility genes, tRNA genes, and flanking direct repeats. In the case of PAIs, that region also contains virulence factor genes. The advantage of this approach is that it relies on only the query genome sequence, and closely-related species genomes are not needed, thus making it possible to predict GIs of all genomic sequences. Below, we describe sequence composition approaches, based on the alphabetic order of programs. It should be noted that all programs, except PIPS, are for GI detection in general. The summary of these programs as well as their websites can be found in Table 2.

AlienHunter is one of popular software packages that use sequence composition-based approach [15]. This key idea of this software is to describe the sequence signatures by using interpolated variable order motifs (IVOM). Specifically, this method exploits compositional biases at various levels by implementing variable order motif distributions, and, thus, it can capture sequence signature accurately with variable length of sequence. AlienHunter focuses on higher order motifs if the gnomic region is long enough, so that it can make accurate prediction results. When the genomic region to be tested is short and the information is not sufficient, it considers lower order motifs. Generally speaking, the higher the IVOM score is the more GI segment the genome sequence looks like.

**Table 2:** The summary of a list of sequence composition based software.

Software ^a^	Main Principle	System Setup ^b^	Website
AlienHunter	HMMs on various mer words	Unix/Linux OS, Java and Perl environment setup	http://www.sanger.ac.uk/resources/software/alien_hunter/
Centroid	Centroid on *k*-mer word	Unix/Linux OS and C++ environment setup	Upon request
EGID	Ensembles the results of AlienHunter, IslandPath, SIGI-HMM, INDeGenIUS and PAI-IDA	Unix/Linux OS, Java, C++ and Perl environment setup	http://www5.esu.edu/cpsc/bioinfo/software/EGID
GIDetector	Decision-tree based bagging on IVOM score, insertion point, size, gene density, repeats, integrase, phage and non-coding RNA	Windows OS, C# with the support of Perl and Cygwin	http://www5.esu.edu/cpsc/bioinfo/software/GIDetector/
GI-GPS	SVMs on sequence composition (including GC content, dinucleotide frequency, codon usage, and codon adaption usage), and with filtering steps including length of candidate segment, tRNA and repeat elements	Not available	http://gipop.life.nthu.edu.tw
GIHunter	Decision tree based bagging model using sequence composition, gene information and inter-genic distance, mobile genes, phage genes, tRNA, and gene density	Unix/Linux OS, Java, C++ and Perl environment setup	http://www5.esu.edu/cpsc/bioinfo/software/GIHunter
INDeGenIUS	Clustering/Centroid on *k*-mer word	Unix/Linux OS and C++ environment setup	Upon request
IslandPath	G+C, dinucleotide, mobile genes, and codon usage	Unix/Linux OS and Perl environment setup	http://www.pathogenomics.sfu.ca/islandpath
PAI-IDA	Discriminant analysis on G+C, dinucleotide and codon usage	Unix/Linux OS, C++ and Perl environment setup	http://compbio.sibsnet.org/projects/pai-ida
PIPS	G+C content, codon usage deviation, virulence factors, hypothetical proteins, transposases, flanking tRNA and its absence in nonpathogenic organisms	Unix/Linux OS and Perl environment setup	http://www.genoma.ufpa.br/lgcm/pips
SIGI-HMM	HMM on codon usage	Unix/Linux OS and Java, environment setup	http://www.tcs.informatik.uni-goettingen.de/colombo

^a^ PIPS is used for predicting PAIs specifically, the rest of software tools are used for predicting GIs in general, including PAIs; ^b^ System setup include the operating systems in which software tools are run, and additional software may be installed such as Java/Perl/C++ environments.

Since AlienHunter predicts islands only based on genomic sequence, not on pre-existing annotation or gene position information, thus it can be applied on the newly sequenced genome. It has been reported that AlienHunter has high prediction sensitivity (*i.e.*, detecting most of actual existing islands), but with high false positives too [49]. One of the causes is that AlienHunter does not exclude the region with highly expressed genes, which also show high IVOM scores.

Centroid [16] is an approach that identifies compositionally distinct regions in genomes using word frequencies. In particular, the query genome is separated into non-overlapping groups of equal length. For any given group, this tool finds the frequencies for all possible words with the length of *m*. Since there are four possible symbols A, C, G, and T, the total number of possible words is *n* = 4*^m^*. The average of each word frequency based on the whole genome can be calculated, and this is considered to be the centroid. The distances between any genomic region and the centroid based on word frequencies are computed. The outliers can be determined based on the distance calculation, and these regions are considered to be the GIs.

EGID [56] is an ensemble algorithm for island detection, which takes the prediction results of existing computational tools (including AlienHunter [15], IslandPath [57], SIGI-HMM [17], INDeGenIUS [58], and PAI-IDA [59]), and then generates consensus results by using voting algorithm. Performance comparisons between this ensemble algorithm and individual programs showed that the ensemble algorithm was better than any other program in terms of prediction sensitivity and specificity.

To make the software user-friendly, Hasan *et al*. [60] later developed Genomic Island Suite of Tools (GIST). GIST provides a platform so that third-party programs were embedded in EGID. GIST also includes a downloadable feature to facilitate collecting genome sequences automatically from the FTP server of the National Center for Biotechnology Information (NCBI).

GIDetector [61] is a J48-based decision tree-bagging model for island prediction. The authors tested different ensemble algorithms including adaBoost, bagging, multiboost, and random forest [62,63], and found bagging was the best classifier model. The model was trained based on the features of IVOM score (collected from AlienHunter [15]), insertion point, size of the genomic region, number of genes per kb, repeats (computed through REPuter [45]), integrase, phage, and non-coding RNA. The program has the feature of collecting genome sequences from public websites directly, and then predict island regions based on the training model.

GI-GPS (Genomic Island Genomic Profile Scanning) [64] is a support vector machines (SVMs) based GI prediction model. This construction of SVMs is based on four categories of feature information, including: (1) codon usage frequency; (2) dinucleotide frequency; (3) codon adaption index; and (4) GC content. The GI-GPS starts with truncating the whole genome into fixed sized segments, with each segment will be classified into potential GI segments using SVMs, and then merges them into large segments, followed by some filtering steps, based on the length of the segment and the existence of Mobile Genetic Elements (MGE). In the final stage, GI-GPS refines the boundaries of predicted GIs by locating the positions of tRNA genes and repeating elements.

GIHunter [43] uses gene information and inter-genic distance along with sequence information to predict genomic islands. This tool uses a training set obtained from 113 genomes and developed a decision tree based bagging model for genomic island prediction. The features of gene information (*i.e.*, highly expressed genes) and inter-genic distance were found to improve the genomic island prediction accuracy, which have not been reported in other studies. The authors recently incorporated the features of phage genes, mobility genes, tRNAs and gene density, and, thus, prediction accuracy was improved further.

INDeGenIUS [58] is a method named as Improved N-mer based Detection of Genomic Islands Using Sequence-clustering (INDeGenIUS). This algorithm basically uses the principles of hierarchical clustering to find the real “centroid”. This tool first divides the query genome into “*n*” overlapping groups of equal size. For each group, the frequencies of word length of “*k*” are computed, and a vector of 4*^k^* words is computed. The word enumeration process for each group, thus, can generate “*n*” clusters. By computing the distances of all possible pairs of groups and using the hierarchical clustering schemes, this tool can iteratively merge groups into some number of clusters. At this stage, the cluster that meets the percentage threshold (in terms of the number of groups) is considered to be “major cluster”, otherwise “minor clusters”. Based on the members of the “major cluster”, this tool finds the real “centroid” of the host genome, and uses it for GI prediction as the original centroid approach.

IslandPath [57] incorporates multiple DNA signals and genome annotation features to predict GIs. Features includes in this approach are: (1) the %G+C of predicted open reading frames; (2) dinucleotide bias for gene-clusters; (3) the location of known or probable mobility genes; (4) the location of tRNAs. The final results of GIs prediction are graphically displayed in this software package, so users have the options to determine if a region is a real GI or not, based on their expertise.

PAI-IDA [59] uses interactive discriminant analysis for GI prediction. In particular, the authors define genomic islands that deviate most from the rest of the genome in three compositional criteria: G+C content, dinucleotide frequency and codon usage. In this tool a small list of known PAIs from seven genomes was used for building up the training dataset. This dataset was used to generate the parameters of the linear functions that extract the anomalous regions from the rest of the genome. The discriminant function is improved through iteration by taking additional predicted anomalous regions into account.

PIPS (Pathogenicity Island Prediction Software) [65] is a software suite designed for predicting pathogenicity islands. This approach uses multiple features in order to predict PAIs. Unlike most of other prediction tools that are used to predict islands in general, this is one of a few tools used for predicting PAIs specifically. Features used in PIPS include atypical G+C content, codon usage deviation, virulence factors, hypothetical proteins, transposases, flanking tRNA, and its absence in nonpathogenic organisms.

SIGI-HMM [17] predicts GIs based on the codon usage bias. It first analyzes the codon usage of each gene, provides the score for each gene based on the codon usage, and thus it can find alien genes based on codon usage scores. The way of finding genomic islands based on codon scores is called SIGI [66]. Later on, the authors also applied Hidden Markov Model (HMM) to SIGI approach to improve GIs prediction. As GIs usually have a considerable length, HMM was implemented to access GI prediction on the gene level. This approach is sensitive for the identification of GIs in microbial genomes.

Out of all software tools introduced above, only PIPS predicts PAI specifically. Other software tools are used to predict all genomic islands, including pathogenicity islands. It should be noted that different software tools were implemented with various computer languages such as C++, Java or perl script, and they might only be executed in certain operating systems such as Linux.

## 4. Databases and Related Computational Resources

Other than those GI prediction software tools discussed above, there are a number of PAI related databases and online resources that can be accessed directly. The online resources will be especially useful for microbiologist and medical scientists who are not familiar with computer languages, and thus find it difficult to execute command line programs under Linux systems. Below, we describe these online databases and servers. The summary of these resources as well as their websites can be found in Table 3.

**Table 3:** The summary of public island databases and web resources.

Category	Description	Website
**GI Databases/Servers**		
DGI	A database that contains genomic islands of more than 2,000 bacterial genomes, many of which are PAIs, and displays GIs in circular graphic images	http://www5.esu.edu/cpsc/bioinfo/dgi
GI-POP	A database that provides ongoing microbial gnome annotation, including ORF annotation, non-coding RNAs and GIs. GIs are predicted using GI-GPS	http://gipop.life.nthu.edu.tw
IGIPT	A web server that identifies islands based on standard deviation from sequence composition average	http://bioinf.iiit.ac.in/IGIPT/
Islander	A database that contains a list of 89 islands in 106 bacterial genomes that harbor tRNA and tmRNA genes, and integrase genes	http://www.indiana.edu/~islander
IslandViewer	A database that contains predicted GI based on IslandPick, IslandPath-DIMOB and SIGI-HMM, and displays GIs in circular graphic images	http://www.pathogenomics.sfu.ca/islandviewer/query.php
MOSAIC	A database that contains conserved segments and various regions (*i.e.*, GIs) in bacterial genomes, predicted by comparative genomic approach	http://genome.jouy.inra.fr/mosaic
**PAI Databases/Servers**		
PAIDB	A database contains known PAIs, candidate PAIs which are homologous to known PAIs	http://www.gem.re.kr/paidb
PredictBias	A web server that calculates PAIs based on %G+C, dinucleotide, codon usage, virulence factor and absence of non-pathogenic species	http://www.davvbiotech.res.in/PredictBias
**Virulence Factor Databases/Servers**		
MvirDB	A database that contains a collection of publicly available and organized sequences representing known toxins, virulence factors, and antibiotic resistance genes	http://mvirdb.llnl.gov/
VFDB	A database that contains all known virulence factors, as well as homologous genes through similarity search	http://www.mgc.ac.cn/VFs/
VirulentPred	A web server that predicts virulence factors based on input protein sequences	http://bioinfo.icgeb.res.in/virulent/

### 4.1. PAI Databases and Servers

DGI (Database of Genomic islands) contains predicated genomic islands of more than 2,000 microbial genomes, including pathogenic bacteria. The genomes were downloaded from the NCBI web server, and predicted by GIHunter program. For each genome, a circular representation of genomic island image was generated by GIV (Genomic Island Visualization) [67], which is basically a customized Circos [68]. Each genomic island image displays the locations of genomic islands, and supportive features including IVOM, HEG, tRNA, gene density, phage, integrase, inter-genic distance, and transposases.

GI-POP [64] is a web-based tool that is used not only for predicting GIs, but also for assembling genome sequences and annotating gene functions. Users can submit draft microbial genomes of the ongoing genome projects in contigs or scaffolds to the GI-POP web server and can get the functional annotation and predicted GI results. GI-POP uses the DIYA assembler [69] to assemble contigs or scaffolds. The annotation pipeline in GI-POP includes: (1) coding region annotations which use the gene finding program such as Glimmer software and COG database; (2) non-coding region annotations using tRNA-scan and RNAmmer; and (3) GI prediction using GI-GPS. GI-GPS is basically a SVMs classifier, described in the previous section. GI-POP provides a number of nice user interfaces such as the feature of allowing multiple users to do online genome annotation and GI prediction.

IGIPT [70] is a web-based integrated platform for GI identification. This tool incorporates thirteen parametric measures, which can be narrowed down into two kinds of signatures: (1) genomic signatures such as G+C content and *k*-mer frequencies; and (2) codon signature. The tool treats the regions to be putative GIs if measured feature values in that region deviate from genomic average. This tool provide an option for downloading the predicted GI and flanking regions so that users study various structural features, such as tRNA, integration sites and repeats. The limitation of this web tool is that it leaves users to decide standard deviation so only users who know reasonable deviation will be able to obtain meaningful GIs. In addition, these thirteen measures are redundant information and might not be additive for prediction.

Islander [71] is a database that contains predicted GI regions from bacterial genomes. The islander algorithm performs the following major steps: (1) identify candidate island regions, which are the sites that contain tRNA and tmRNA genes (considered to be the end points of islands), searched by tRNAScan-SE [36] and BRUCE [72]; (2) search the regions that contain integrase genes using Pfam database [28]; and (3) combine candidate island regions with the regions containing integrase genes, which are considered as GI regions. Out of 106 bacterial genomes, the authors identified 143 candidate islands. Among the candidate islands, regions without integrase genes were filtered out and finally 89 islands were stored in the database.

IslandViewer [73] contains pre-computed GI predictions in all sequenced bacterial and Archaeal genomes. GIs are predicted using three popular prediction tools, IslandPick [49], IslandPath-DIMOB [37], and SIGI-HMM [17]. The graphical interface allows users to easily view and download the island data in multiple formats, at both the chromosome and gene level. The web-server is updated automatically on a monthly, so new sequenced gnomes can be predicted and uploaded to the website in time. In addition, users can upload their own genome sequence for GI prediction. A recent update of IslandViewer [74] has been released to facilitate custom genome analyses in a better fashion. This new version provides additional gene information including virulence factors, antibiotic resistance genes and pathogen-associated genes. This tool also shows the comparisons of GIs between user-selected genomes through a side-by-side viewer.

PAIDB [75] is a comprehensive database that contains three kinds of PAIs: (1) previously reported PAIs, which were obtained through literature search; (2) candidate PAIs (cPAIs), which are homologous to known PAIs and overlap with predicted GIs; and (3) non-probable PAIs (nPAIs), which are homologous to known PAIs but do not overlap with predicted GIs; The authors previously developed a pathogenicity island search tool PAI Finder [76] to identify cPAIs and nPAIs. PAI Finder first identifies PAI-like regions homologous to known PAIs using BLAST, and then uses %G+C and codon usage to detect GI regions. The overlapped regions are treated as cPAIs. PAIDB also provide functionalities to allow users to search for PAI regions by species, by text and also by using BLAST. To our best knowledge, PAIDB is the only database that contains annotated PAIs and predicted ones. Unfortunately, this database has not been updated since the release of PAIDB in 2006. Another main limitation of this database is that novel PAIs were excluded as candidate PAIs were homologous to previously described PAIs in the literature.

PredictBias [77] is a web server for predicting GI and PAI regions in prokaryotic genomes. The authors used the features of sequence composition bias (*i.e.*, %GC, dinucleotide and codon), virulence associated genes, and absence in related non-pathogenic species for their prediction. PredictBias first predicts GI regions using sequence composition biased information, and then compares the regions with entries of Virulence Factor Profile Database (VFPD). If any of the predicted regions shows significant composition bias and encodes at least one of the proteins listed in VFPD, then that region is considered as potential PAI (biased composition). If regions containing unsuspicious composition bias but harboring more than four VFPD proteins, then it can be considered as unbiased composition potential PAIs. By using “compare genome feature” to confirm the absence of potential PAIs in non-pathogenic species, users can confirm the validity of identified potential PAIs.

### 4.2. Virulence Factor Databases and Servers

MvirDB [24] hosts a collection of known toxins, virulence factors, and antibiotic resistance genes. This collection facilitates the rapid identification of sequences and characterization of genes for signature discovery, which is useful for the community involved in bio-defense research. The original data source were obtained from eight databases: Tox-Prot [78], SCORPION [79], PRINTS virulence factors [80], VFDB [81], TVFac toxin and virulence factor database, Islander [71], ARGO [82], and VIDA [83]. MvirDB data are maintained through microbial annotation database (MannDB) system. MvirDB provides users the features of querying a DNA or protein sequences using BLAST search from the database. In addition, the browser tool allows users to retrieve description, sequences and classification of virulence factors. MvirDB is automatically updated each week so that novel discovered genes and proteins can be deposited in the database. MvirDB has been used for finding virulence factors in one of the PAI software tools of PIPS [65].

VFDB [81,84] is an integrative and comprehensive database of virulence factors from bacterial pathogenes. This database provides detail information such as structure features, function and mechanisms of known virulence factors. Known virulence factors in VFDB were collected through literature search of verified one on PubMed, and putative virulence factor liked genes are also stored in the database. VFDB allows users to browse this database by species, text search, BLAST, and PSI-BLAST. In a later version of VFDB [84], released in 2008, some new features were provided, including tabular comparison of pathogenomic composition in terms of virulence, multiple alignments and statistical analysis of homologous virulence genes, and graphical comparison of pathogenomic organization of virulence factors. In the most recent version of VFDB 2012 [85], more user interfaces such as expanded trees, collapsible menus, and tabbed panels have been added. These new features could help researchers do inter-genera comparative analysis of VFs, and, thus, to further understand the evolutions of VFs. VFDB has been used in a number of PAI detection approaches and web resources, including IslandViewer [74], PAIDB [75], and PredicBias [77].

VirulentPred [25] is a two-layer SVM based prediction tool for virulence factors. The virulentPred model was trained based on known virulence factors collected from SWISS-PROT [86] and VFDB [81]. The first layer SVM model was trained based on features of amino acid composition, dipeptide composition, and high-order dipeptide composition. The second layer SVM model was trained based on the SVM score from the first layer, and PSI-BLAST result. The accuracy of VirulentPred was reported to be as high as 82% from the experiments. The advantage of using VirulentPred is that we can discover potential virulence factors that were found in our current virulence factor database.

## 5. Concluding Remarks

We have reviewed existing computational approaches for PAI detection in bacterial genomes. For comparative genomics approaches, the query genome is aligned with its phylogenetically closely-related genomes, and unique acquired regions with virulence factors were predicted to be PAIs. For sequence composition based approaches, one or multiple PAI-associated features are applied, and then various scoring functions, such as HMMs and decision-tree-based ensembles, are used to evaluate these genomic regions based on their feature values. Additionally, we have provided a list of PAI related web resources for scientific community to access.

It should be noted that there is no systematic performance evaluation on current PAI prediction tools, though each of these methods was evaluated in a limited number of genomes. Independent evaluations of prediction tools have been designed and performed in other bioinformatics areas, such as the evaluation for motif discovery tools [87], and the evaluation for operon prediction tools [88]. It might be useful to collect a number of independent known PAIs from previous studies, and evaluate all existing PAI prediction tools in a similar fashion.

Additionally, using sequence based computational approaches cannot find all existing PAIs. They might not be able to identify the PAIs whose sequence composition is similar to that of the core genome. They cannot find PAIs acquired long time ago either, because amelioration can make the sequence composition (or codon usage) of the PAIs be similar to that of the core genome. The limitation of sequenced based approach can be complemented by using comparative genomic approach, which does not compare sequence compositions of the potential PAIs and those of the core genome. However, comparative genomic approaches themselves have their shortcomings, *i.e.*, they need phylogenetically closely-related reference genomes for any query genome. Therefore, both approaches have pros and cons, and it might be beneficial to use both approaches to identify all existing PAIs.

The idea of the integration of comparative genomic and sequence based approaches for reliable GI identification has been used in IslandViewer [73,74] to some degree. However, the users’ must make their own decisions to select which predicted ones from multiple programs are reliable. Furthermore, there is no indication which predicted GIs by comparative genomic approach have similar sequence composition with the core genome, and thus the advantage of using comparative genomic approach becomes minimal. In the future work, an integrated version of PAI software tool can be developed similar to IslandViewer, but with the incorporation of pathogenic-associated gene information, and the feature of automatic evaluations of predicted results, which can be implemented similar to the development of EGID [56].

While there are a number of review papers summarizing a list of discovered PAIs in various pathogenic bacteria, there is only one annotated PAI database dated back to 2006, and there is no update since then. It will be useful to build a comprehensive annotated PAI database, which will host all discovered PAIs to this date. The construction of such databases might also improve computational prediction tools by reevaluating the PAI-associated features based on discovered PAIs. For example, we can have better understanding of which genomes prefer what kinds of tRNA genes after doing statistical analyses of tRNA genes in discovered PAIs.

Finally, the development of computational approaches for finding the relationship between donor and recipient genomes through PAIs might also be very useful. The corresponding visualization tools for revealing their relationships might be needed so that researchers can use them to study gene transfer mechanisms.
